# RESEARCH FOR ACTION: IDENTIFYING SERVICE NEEDS OF MONGOLIAN OLDER ADULTS

**DOI:** 10.1093/geroni/igz038.3483

**Published:** 2019-11-08

**Authors:** Uyanga Batzogs, Kathryn Braun, Margaret Perskinson, Tyran M Terada

**Affiliations:** University of Hawaii, Honolulu, Hawaii, United States

## Abstract

Although adults aged 60 years and older currently represent 6% of the total population of Mongolia, they are projected to increase to 19% by 2050. More than 21% of Mongolians live below the poverty line. Social security payments represent the main source of income for many retirees. With little government funding, eldercare services are limited, creating a large gap between service needs and availability. Before an effective system of eldercare can be developed, in-depth understanding of older adults’ needs and resources is required. A mixed methods design was used. Four-hundred twenty-seven Mongolians aged 55 years and older were surveyed. Two focus groups with 10 older adults and eight in-depth interviews with senior center stakeholders were conducted between June 2019 and August 2019. Descriptive statistics were run to determine frequencies of participants’ service needs. Linear regression examined the relationship between age groups, service needs, and ability to pay for services. Focus group and interview transcripts were analyzed for underlying themes. Findings indicated high service needs among older adults. Retirement homes, assisted living, home care, and places to socialize were identified as the most needed services. Lack of services, employment opportunities, and income sources concerned them the most. Ability to pay for services was negatively correlated with age groups. Qualitative analysis yielded two themes: importance of services and lack of resources. Further research on starting and sustaining networks of supportive services for older adults living in Mongolia is needed.

